# TRPA1 Ion Channel Determines Beneficial and Detrimental Effects of GYY4137 in Murine Serum-Transfer Arthritis

**DOI:** 10.3389/fphar.2019.00964

**Published:** 2019-09-04

**Authors:** István Z. Bátai, Cecília Pápainé Sár, Ádám Horváth, Éva Borbély, Kata Bölcskei, Ágnes Kemény, Zoltán Sándor, Balázs Nemes, Zsuzsanna Helyes, Anikó Perkecz, Attila Mócsai, Gábor Pozsgai, Erika Pintér

**Affiliations:** ^1^Department of Pharmacology and Pharmacotherapy, Medical School and János Szentágothai Research Centre & Centre for Neuroscience, University of Pécs, Pécs, Hungary; ^2^Department of Organic and Pharmacological Chemistry, Medical School, University of Pécs, Pécs, Hungary; ^3^Department of Medical Biology, Medical School, University of Pécs, Pécs, Hungary; ^4^Department of Physiology, Faculty of Medicine, Semmelweis University, Budapest, Hungary; ^5^MTA-SE “Lendület” Inflammation Physiology Research Group of the Hungarian Academy of Sciences and the Semmelweis University, Budapest, Hungary

**Keywords:** GYY4137, TRPA1, K/BxN serum-transfer model, arthritis, hydrogen sulphide, polysulfide, hypochlorite, MIP-2

## Abstract

Modulation of nociception and inflammation by sulfide in rheumatoid arthritis and activation of transient receptor potential ankyrin 1 (TRPA1) ion channels by sulfide compounds are well documented. The present study aims to investigate TRPA1-mediated effects of sulfide donor GYY4137 in K/BxN serum-transfer arthritis, a rodent model of rheumatoid arthritis. TRPA1 and somatostatin sst4 receptor wild-type (WT) and knockout mice underwent K/BxN serum transfer and were treated daily with GYY4137. Functional and biochemical signs of inflammation were recorded, together with histological characterization. These included detection of hind paw mechanical hyperalgesia by dynamic plantar esthesiometry, hind paw volume by plethysmometry, and upside-down hanging time to failure. Hind paw erythema, edema, and passive movement range of tibiotarsal joints were scored. Somatostatin release from sensory nerve endings of TRPA1 wild-type and knockout mice in response to polysulfide was detected by radioimmunoassay. Polysulfide formation from GYY4137 was uncovered by cold cyanolysis. GYY4137 aggravated mechanical hyperalgesia in TRPA1 knockout mice but ameliorated it in wild-type ones. Arthritis score was lowered by GYY4137 in TRPA1 wild-type animals. Increased myeloperoxidase activity, plasma extravasation, and subcutaneous MIP-2 levels of hind paws were detected in TRPA1 knockout mice upon GYY4137 treatment. Genetic lack of sst4 receptors did not alter mechanical hyperalgesia, edema formation, hanging performance, arthritis score, plasma extravasation, or myeloperoxidase activity. TRPA1 WT animals exhibited smaller cartilage destruction upon GYY4137 administration. Sodium polysulfide caused TRPA1-dependent somatostatin release from murine nerve endings. Sulfide released from GYY4137 is readily converted into polysulfide by hypochlorite. Polysulfide potently activates human TRPA1 receptors expressed in Chinese hamster ovary (CHO) cells. According to our data, the protective effect of GYY4137 is mediated by TRPA1, while detrimental actions are independent of the ion channel in the K/BxN serum-transfer arthritis model in mice. At acidic pH in inflamed tissue sulfide is released from GYY4137 and reacts with neutrophil-derived hypochlorite. Resulting polysulfide might be responsible for TRPA1-mediated antinociceptive and anti-inflammatory as well as TRPA1-independent pro-inflammatory effects.

## Introduction

Despite a heavy improvement in remissions and disease activity due to emerging targeted disease-modifying biologic treatment options, adverse drug reactions still make the pharmacotherapy of rheumatoid arthritis (RA) a challenging quest for patient and physician (Chatzidionysiou et al., 2017; Edwards et al., 2017). Animal models of the disease are valuable tools in the search for therapeutic targets. The murine K/BxN serum-transfer arthritis is a much-preferred model of RA because it is suitable for a variety of gene-deficient mouse strains and is extremely reliable. Arthritis is induced by intraperitoneal administration of serum from genetically arthritic K/BxN animals. Development of inflammatory response involves autoantibody formation against the enzyme glucose-6-phosphate isomerase and immune complex-driven activation of neutrophil granulocytes and macrophages. Several cytokines, chemokines, the complement system, adhesion molecules, and receptors contribute to the molecular pathology. Although glucose-6-phosphate isomerase is surely not the major autoantigen in human RA, the K/BxN serum-transfer arthritis model is very useful in the investigation of the interaction between autoantibodies and the innate immune system, as well as inflammatory pain (Christensen et al., 2016).

The gaseous mediator hydrogen sulfide (in the following text, the term sulfide denotes H_2_S, HS^−^, and S^2−^) is known to play a regulatory role in inflammation. Some authors report anti-inflammatory effects of H_2_S, while others have found pro-inflammatory action (Burguera et al., 2017; Muniraj et al., 2017). The two kinds of findings might be both true and complementary. Inflammation might induce the H_2_S-producing enzyme cystathionine-γ-lyase (CSE) resulting in elevated H_2_S concentration. H_2_S might contribute to inflammatory vasodilatation and might compromise mitochondrial function by inhibiting complex IV of the electron transport chain. On the other hand, smaller H_2_S concentration in the mitochondria can actually fuel oxidative phosphorylation and mitigate oxidative stress (Ahmad and Szabo, 2016). Today, slow-releasing donors of sulfide are preferred over inorganic sulfide sources (e.g., NaHS and Na_2_S-nonahydrate). Inorganic salts produce a swift but short-lived elevation of sulfide concentration not necessarily representing physiological conditions. Slow-releasing donors might better mimic the endogenous situation. The two most studied donors are GYY4137 and AP39. GYY4137 releases sulfide in every tissue, while AP39 is selective to mitochondria, enabling the investigation of the protective effects of the gasotransmitter (Li et al., 2008; Szczesny et al., 2014).

Transient receptor potential ankyrin 1 (TRPA1) non-selective cation channel belongs to transient receptor potential ankyrin receptor subfamily of transient receptor potential ion channels. It is the sole ankyrin-type TRP channel in mammals. TRPA1 is expressed in primary nociceptor neurons and non-neuronal cells including joint-related cells (e.g., chondrocytes and synoviocytes) and immune cells (Zygmunt and Högestätt, 2014). TRPA1 is known to contribute to inflammatory hyperalgesia in murine complete Freund’s adjuvant-induced (CFA) arthritis—another animal model of human RA (Fernandes et al., 2016; Horváth et al., 2016). Expression of TRPA1 channels in peripheral blood leukocytes of RA patients was shown to correlate with the perceived pain (Pereira et al., 2017). TRPA1 receptors can be activated by various stimuli ranging from temperature to chemicals. Electrophilic agents excite TRPA1 by forming covalent bonds with cysteine residues of the receptor (Zygmunt and Högestätt, 2014). Sulfide is able to activate TRPA1 receptors (Hajna et al., 2016). This interaction is most probably transmitted by polysulfides—originating from sulfide—in concert with hypochlorite or nitric oxide (Greiner et al., 2013; Cortese-Krott et al., 2015). These characteristics set sulfide in the focus point of the modulation of the inflammatory process in RA, marking it as a promising drug target. A well-characterized potential downstream mechanism of activation of peptidergic nociceptors via TRPA1 opening is a consequent release of somatostatin (SOM) from the nerve fibers and signaling of sst4 receptors on nociceptors and other cell types (Pintér et al., 2006).

The present study was intended to investigate the role of sulfide and TRPA1 channels in the murine K/BxN arthritis model. The slow-releasing donor of sulfide, GYY4137, was administered to TRPA1 wild-type (WT) and knockout (KO) animals undergoing serum-evoked polyarthritis. Functional parameters of the arthritic changes (e.g., mechanical hyperalgesia and grip strength) were evaluated alongside with markers of inflammation (e.g., clinical score, plasma extravasation, myeloperoxidase (MPO) activity, and cytokine concentrations in the affected joints). Similar experiments were conducted with mice lacking somatostatin sst4 receptor gene. Release of SOM from the isolated skin pieces of TRPA1 WT and KO animals due to stimulation with polysulfide (POLY) was detected by radioimmunoassay. Formation of POLY from GYY4137 upon reaction with hypochlorite was characterized by cold cyanolysis.

## Materials and Methods

### Animals

Experiments were carried out on male TRPA1 or sst4 deficient mice (KO) and on their wild-type counterparts (WT). Both strains were generated on C57BL/6J background. The experimental animals were offspring of heterozygous parents, and their genotype was determined using real-time polymerase chain reaction (PCR) technique. The original heterozygous TRPA1 breeding pair was a generous gift from Pierangelo Geppetti (University of Florence, Italy). These mice were originally generated and characterized by Bautista and colleagues (Bautista et al., 2006). Sst4 KO animals were generated by Jeremy P. Allen and Piers C. Emson (Helyes et al., 2009). The mice were kept and bred in the Laboratory Animal House of the Department of Pharmacology and Pharmacotherapy at the University of Pécs under standard pathogen-free conditions. Standard mouse chow and water were provided *ad libitum*. Temperature was maintained between 24°C and 25°C, and the light–dark cycle was set to 12 h. Mice were housed in groups of 5–10 in polycarbonate cages (330-cm^2^ floor space, 12-cm height) on wood shavings bedding. Certificate numbers of the animal breeding facilities (*Department of Pharmacology and Pharmacotherapy and Szentágothai Research Centre, University of Pécs*) are ZOHU0104L09 and ZOHU0104L03.

### Drugs and Chemicals

All chemicals were purchased from Sigma-Aldrich, except for the following: ketamine (Calypsol, Gedeon Richter Plc., Budapest, Hungary), xylazine (Sedaxylan, Eurovet Animal Health B.V., Bladel, the Netherlands), luminol sodium salt (Gold Biotechnology, Olivette, MO, USA), and IR-676 (Spectrum-Info Ltd., Kyiv, Ukraine). GYY4137 was synthetized at the Department of Organic Chemistry, Medical School, University of Pécs, Pécs, Hungary, as previously described (Li et al., 2008).

### Detection of Polysulfide Formation From GYY4137 by Cold Cyanolysis

GYY4137 was dissolved in distilled water (7.95 mmol L^−1^), and equal volume of citrate buffer (100 mmol L^−1^, pH 3.0) was added. The mixture was incubated for 90 min, and equal volume of hypochlorous acid (200 μmol L^−1^) was added. Cold cyanolysis was performed as described earlier (Wood, 1987). The mixture of hypochlorous acid and GYY4137 was alkalized and reacted with KCN. Formaldehyde and Goldstein reagent (Bátai et al., 2018) were added, and absorbance was detected at 460 nm. POLY concentration was calculated according to a standard curve constructed with KSCN.

### Measurement of Ca^2+^ Influx Into CHO Cells Expressing Human TRPA1 in Response to GYY4137 and Hypochlorous Acid by Flow Cytometry

Chinese hamster ovary (CHO) cells were transfected with human TRPA1, as described earlier (Pozsgai et al., 2017). Human TRPA1 cDNA clone was purchased from Life Technologies CloneRanger collection (clone ID: 100016279). The cDNA was inserted between the cytomegalovirus (CMV) promoter and the bovine growth hormone polyA region in pT3CMV vector. pT3CMV originates from the pT2/BH Sleeping Beauty transposon vector (Addgene, ID: 26556) with neomycin expression cassette. CHO cells were co-transfected with vectors containing human TRPA1 and the pCMVSB100x vector carrying the transposase. Successful clones were selected in medium containing G418 (500 μg mL^−1^). After trypsin treatment, cells were loaded with Fluo-4 AM (Invitrogen) for 30 min at 37°C. Extracellular solution (ECS) was added (400 μL, containing in mmol L^−1^: NaCl, 160; KCl, 2.5; CaCl_2_, 1; MgCl_2_, 2; HEPES, 10; glucose, 10; pH 7.3). Sodium sulfide nonahydrate (Na_2_S·9H_2_O) was dissolved in Millipore water. Tubes used were previously filled with nitrogen. Concentration of the solution was measured by detecting light absorption at 230 nm (based on *E*_230_ = 7,700 L mol^−1^ cm^−1^), as well as adding 5,5′-dithiobis(2-nitrobenzoic acid) and measuring light absorption at 412 nm. A concentration response curve was constructed using various concentrations of sodium sulfide nonahydrate. GYY4137 was dissolved in distilled water (3 mg mL^−1^, 7.95 mmol L^−1^). GYY4137 was diluted further in ECS buffer, and a concentration–response curve was produced. POLY (10 μmol L^−1^ in ECS) was added to the cell suspensions, too. POLY was prepared as described earlier (Bátai et al., 2018). Stock solutions of hypochlorous acid and sodium sulfide nonahydrate were prepared in distilled water using polypropylene tubes blown with nitrogen gas. Stock solutions were aliquoted and stored at −20°C. Sulfide stock solution was diluted in distilled water to 60 mmol L^−1^. Hypochlorous acid solution was added slowly to produce 20 mmol L^−1^ concentration. The resulting POLY solution was diluted two-fold in distilled water containing 4.17% v/v 10× concentrated phosphate-buffered saline (PBS, pH 7.4). Concentrated hydrochloric acid was then added gradually to set the pH to 7.4. This POLY solution was diluted further in ECS buffer. Sulfide and POLY solutions were kept on ice and were protected from direct light. We examined if polysulfide resulting from the reaction GYY4137-released sulfide and hypochlorous acid activates TRPA1 ion channels in CHO cells. Allyl isothiocyanate (100 μmol L^−1^) was used as positive control. GYY4137 was dissolved in distilled water (3 mg mL^−1^, 7.95 mmol L^−1^). Equal volume of hydrochloric acid (1 mmol L^−1^) was added, and the mixture was incubated at room temperature in the dark for 120 min. Sulfide released from GYY4137 was determined with 5,5′-dithiobis(2-nitrobenzoic acid) as described above. Hypochlorous acid (616 μmol L^−1^ final concentration in the mixture) was added to convert sulfide into POLY. Respective concentrations of hypochlorous acid and GYY4137 were tested separately in TRPA1-expressing CHO cells. The mixture of acidified GYY4137 and hypochlorous acid was examined using CHO cells expressing human TRPA1 ion channels and untransfected ones. Cell suspensions were analyzed by flow cytometry. Fluo-4 AM was excited at 488 nm, and fluorescence was detected at 504 nm. Mean green fluorescence of the samples was compared with base fluorescence of dye-loaded control cells, resulting in a baseline value of 1. Some cell groups were pre-incubated with selective TRPA1 receptor antagonist HC-030031 (50 μmol L^−1^ in ECS) for 5 min. Flow cytometry experiments were performed at room temperature.

### Detection of Somatostatin Release From Murine Nerve Endings by Radioimmunoassay

POLY was prepared as described above (Nagy and Winterbourn, 2010; Bátai et al., 2018). POLY solution was diluted in distilled water to yield 10 μmol L^−1^ in the organ bath.

TRPA1 WT and KO mice were sacrificed by cervical dislocation. Hind legs were shaved by a fine clipper. The skin was removed and placed in oxygenated synthetic interstitial fluid (SIF; composition in mmol L^−1^: NaCl 107.8, NaHCO3 26.2, sodium gluconate 9.64, sucrose 7.6, glucose 5.05, KCl 3.48, NaH_2_PO_4_ 1.67, CaCl_2_ 1.53, and MgSO_4_ 0.69) at room temperature. Skin samples were fixed inside-out on acrylic rods by sutures and were transferred to 37°C oxygenated SIF solution in a shaking bath. After being shaken for 30 min, samples were transferred into glass test tubes containing 800 μL of SIF for 5 min. Then samples were forwarded into a new series of similar tubes containing 10 μmol L^−1^ of POLY in SIF for 5 min, and previous incubating solutions were harvested. Finally, the samples were placed into other tubes containing 800 μL of SIF for another 5 min. POLY-containing and final incubating solutions were harvested, too. The solutions of the first series were evaluated for basal, the ones from the second series for stimulated, and the ones of the third series for post-stimulated SOM release. In some experiments, stimulated and post-stimulated fractions contained 50 μmol L^−1^ of HC-030031 (Pozsgai et al., 2017). Wet weight of skin samples was taken after experiments. Radioimmunoassay was performed to detect somatostatin-like immunoreactivity in the incubating solutions as described earlier (Pozsgai et al., 2017). Shortly, phosphate buffer (pH 7.4, 50 mmol L^−1^) containing sodium chloride (100 mmol L^−1^), sodium azide (0.05% w/v), and bovine serum albumin (0.25% w/v) was used for the assay. The assay includes samples or standard peptides, tracer, and antiserum. Somatostatin-14 was used as standard. Tracer was produced from (Tyr1)-somatostatin-14 by iodinating the peptide with Na^125^I. Iodination is catalyzed by 1,3,4,6-tetrachloro-3α,6α-diphenylglycouril. Antiserum has been raised in sheep against somatostatin-14–bovine thyroglobulin. Antiserum was diluted 1:260,000. Immune complexes and free peptides were separated by Norit-A (10% w/v), dextran (molecular weight [MW]: 65–73 kDa, 1% w/v), and commercial fat-free milk powder (0.2% w/v) in distilled water. Radioactivity of both free peptides and immune complexes were detected (Gamma NZ-310, Hungary). Standard curve was generated by plotting the ratio of the activity of pellets and activity of supernatants of standard samples. Results are expressed as total SOM-like immunoreactivity (fmol SOM-LI) per milligram wet skin. Total SOM-LI is made up of the sum of stimulated and post-stimulated values with basal release values subtracted from each value.

### *In Vivo* Luminescence and Fluorescence Imaging

In the K/BxN serum-transfer arthritis model, i.p. luminol sodium salt (5-amino-2,3-dihydro-1,4-phthalazine-dione; 150 mg kg^−1^) dissolved in sterile PBS (30 mg mL^−1^) was used to detect production of reactive oxygen species (ROS) correlated with neutrophil MPO activity. Furthermore, i.v. IR-676 (0.5 mg kg^−1^) fluorescent dye dissolved in aqueous solution of Kolliphor HS 15 (5% v/v) was used to assess plasma protein extravasation 0, 2, and 6 days following serum administration (Botz et al., 2014). Mice were anesthetized by ketamine/xylazine (120/12 mg kg^−1^ i.p.). Bioluminescence was measured for 10 min and fluorescence 20 min postinjection using the IVIS Lumina II (PerkinElmer, Waltham, USA; 120-s acquisition, F/stop = 1, binning = 8 in bioluminescence; auto acquisition time, F/stop = 1, binning = 2, excitation/emission filter 640/700 nm in fluorescence). Living Image*^®^* software (PerkinElmer, Waltham, USA) was used. Identical region of interests (ROIs) were drawn around both ankle joints; then calibrated units of luminescence (total radiance = total photon flux s^−1^) and fluorescence (total radiant efficiency = (photons s^−1^)(ခμW cm^−2^)^−1^) originating from the ROIs were analyzed (Borbély et al., 2015).

### K/BxN Serum-Transfer Model of Autoimmune Arthritis

**Figure 1:**
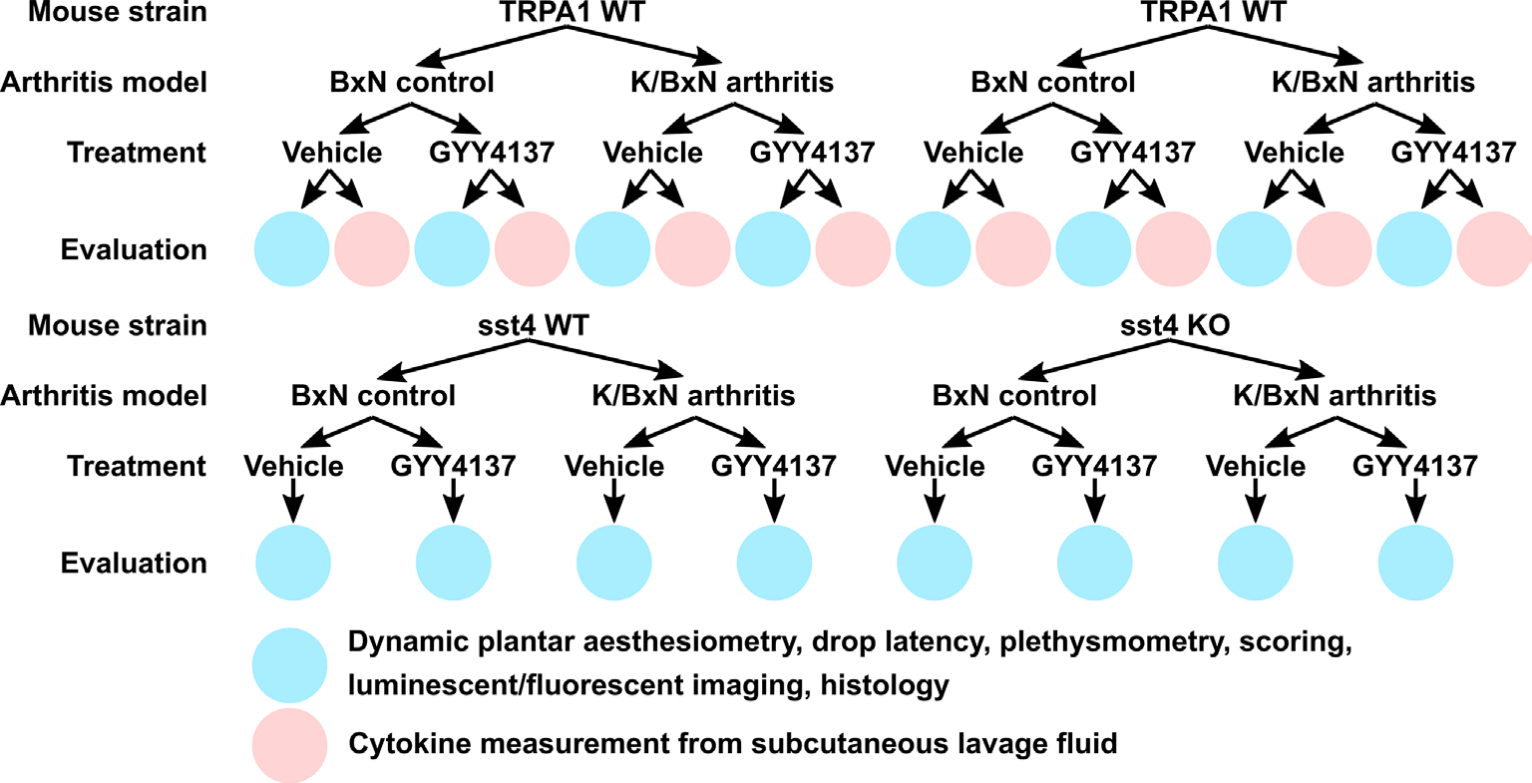
Schematic representation of experimental animal groups and treatments of the study.

Kouskoff et al. generated the K/BxN spontaneously arthritic mouse strain with distinct features representing human rheumatoid arthritis (Kouskoff et al., 1996). Transferring sera from K/BxN mice into non-arthritic mouse strains results in a similar arthritic condition as described in the donor K/BxN strain. In a comprehensive review, Christensen et al. summarized the underlying pathogenesis and distinct features of the K/BxN serum-transfer arthritis model (Christensen et al., 2016). Briefly, in our protocol, serum-transfer arthritis was induced by a single intraperitoneal injection of 300 μL of serum acquired from K/BxN mice. The non-arthritogenic control serum was obtained from BxN mice. Prior to the induction of arthritis, mice received a single treatment of slow-releasing sulfide donor GYY4137 or PBS. The treatment with GYY4137 (50 mg kg^−1^ day^−1^) or PBS was continued daily for 7 days after serum transfer. The intraperitoneal administration of GYY4137 or vehicle always preceded the scoring, functional tests, and *in vivo* imaging by 1 h. The following experimental animal groups were examined: 1) K/BxN serum-treated arthritic TRPA1 WT mice injected daily with vehicle of GYY4137 (PBS) participating in dynamic plantar esthesiometry, drop latency testing, plethysmometry, scoring, and luminescence and fluorescence imaging to detect myeloperoxidase activity and plasma extravasation, as well as histological processing of tibiotarsal joints; 2) TRPA1 KO mice receiving K/BxN serum, identical vehicle treatment, and evaluation; 3) K/BxN serum-injected sst4 receptor WT animals administered vehicle undergoing the above experimental processes; 4) sst4 KO mice treated with K/BxN serum and vehicle used in similar experiments; 5) K/BxN serum-treated arthritic TRPA1 WT mice injected daily with GYY4137 (50 mg kg^−1^ day^−1^) undergoing dynamic plantar esthesiometry, drop latency testing, plethysmometry, scoring, and luminescence and fluorescence imaging to detect myeloperoxidase activity and plasma extravasation, as well as histological processing of tibiotarsal joints; 6) TRPA1 KO animals treated with K/BxN serum and GYY4137 (50 mg kg^−1^ day^−1^) participating in the above experiments; 7) K/BxN serum-injected sst4 receptor WT mice receiving GYY4137 (50 mg kg^−1^ day^−1^) and used in the above experimental settings; 8) K/BxN serum-treated sst4 KO animals injected with GYY4137 (50 mg kg^−1^ day^−1^) undergoing identical processes; 9) K/BxN serum-injected TRPA1 WT mice administered vehicle of GYY4137 (PBS) daily utilized to collect subcutaneous lavage fluid from inflamed hind paws for cytokine detection; 10) TRPA1 KO mice treated with K/BxN serum and vehicle used to collect lavage fluid; 11) K/BxN serum-injected TRPA1 WT mice administered GYY4137 (50 mg kg^−1^ day^−1^) used to collect subcutaneous lavage fluid from inflamed hind paws for cytokine detection; 12) K/BxN serum-treated TRPA1 KO mice administered GYY4137 used to collect lavage samples of the hind paws; and 13–24) the above treatments were performed on non-arthritic control animals injected with BxN serum to validate the serum-transfer arthritis model. **Figure 1** gives a schematic representation of the treatment groups.

### Dynamic Plantar Esthesiometry

Mechanical hyperalgesia of the hind paws was determined by dynamic plantar aesthesiometry (DPA, Ugo Basile 37400, Comerio, Italy). The measurements were performed on three separate days prior to induction of serum-transfer arthritis and thereafter on days 3, 5, and 7. A maximum force equivalent to 10 g with a 4-s latency was applied to the hind paws. Mechanical hyperalgesia was defined as decrease of mechanical pain threshold compared with baseline (average of the three measurements before induction of arthritis) and was expressed in grams.

### Drop Latency/Grip Test

To assess the function of the joints and the overall stamina of the animals, a simple grip test was performed on days 3, 5, and 7. Mice were placed on a wire grid, which was then lifted up in horizontal position and rotated 180° around its horizontal axis, leaving the animal in an upside-down position and forcing it to hold onto the grid against its bodyweight. Healthy mice are able to cling to the grid for at least 20 s. The test was stopped at 60 s. Baseline was acquired during three measurements performed on separate days prior to the induction of serum-transfer arthritis.

### Detection of Hind Paw Volume by Plethysmometry

Volumes of the hind paws were measured by plethysmometry (Ugo Basile Plethysmometer 7140, Comerio, Italy). Both mechanical pain threshold and paw volume measurements were carried out on the same days: three times prior to the induction of arthritis and on days 3, 5, and 7 thereafter. Esthesiometry was always performed first followed by plethysmometry. Baseline volume is the average of three measurements from before the induction of arthritis. Data are expressed in cubic centimeters.

### Arthritis Score

The extent of edema formation and hyperemia of the hind limbs were evaluated on days 3, 5, and 7 according to a semiquantitative clinical score system (0–1.5, healthy condition; 1.5–2.5, minimal signs of disease; 2.5–4, mild inflammation; 4–7, moderate inflammation; and 7–10, severe inflammation) (Jakus et al., 2010; Botz et al., 2014).

### Detection of Inflammatory Cytokines

Cytokine measurements were performed on samples of subcutaneous flush fluid acquired from hind paws as described by Kovács et al. (2014). Briefly, after induction of arthritis, on day 3, mice were anesthetized with ketamine/xylazine (100/10 mg kg^−1^) and were sacrificed via cervical dislocation. Subcutaneous tissue of the hind paws was flushed immediately with 1 mL of ice-cold PBS solution supplemented with 10 mmol L^−1^ of EDTA (pH 7.5) and 20 mmol L^−1^ of HEPES (pH 7.4). Samples were then snap frozen in liquid nitrogen and were stored at −80°C. Quantitative determination of IL-1β, KC, MIP-1α, and MIP-2 concentrations from the samples were performed using MILLIPLEX MAP Mouse Cytokine/Chemokine Magnetic Bead Panel—Immunology Multiplex Assay (Merck Millipore, Billerica, MA, USA). A Luminex 100 device was used for the measurement, and the interpretation of data was performed with the Luminex 100 IS software.

### Histological Evaluation

Tibiotarsal joints were harvested on day 7. Samples were fixed in 4% buffered paraformaldehyde and then decalcified and embedded in paraffin. Sections of 3–5 μm were produced and stained with hematoxylin and eosin (Szabo et al., 2005). Histopathological changes were scored (0–3) by a blinded independent expert. Factors taken into consideration were the following: cartilage destruction, mononuclear cell infiltration, synovial cell proliferation, fibroblast number, and collagen deposition. A composite arthritis score (ranging 0–12) was created (Botz et al., 2014).

### Statistical Analysis

Data are presented as mean ± standard error of the mean. Results were analyzed by one-way analysis of variance (ANOVA) followed by Tukey’s, Dunnett’s, or Bonferroni’s test. Data of SOM release were analyzed by Kruskal–Wallis test due to deviation from normal distribution in various tests. Scatterplots of data on mechanical pain threshold, arthritis score, suspension time, and hind paw volume show all individual data points. Box plots on histological scores show median values (horizontal line), minimal and maximal values (whiskers), and the 25th and 75th percentiles (box). Histological score values were analyzed by Kruskal–Wallis test followed by Dunn’s test. Concentration–response curves were fitted with GraphPad Prism 5.0 using a four-parameter equation.

## Results

### Addition of Hypochlorous Acid to GYY4137 Leads to Formation of Polysulfide

**Figure 2:**
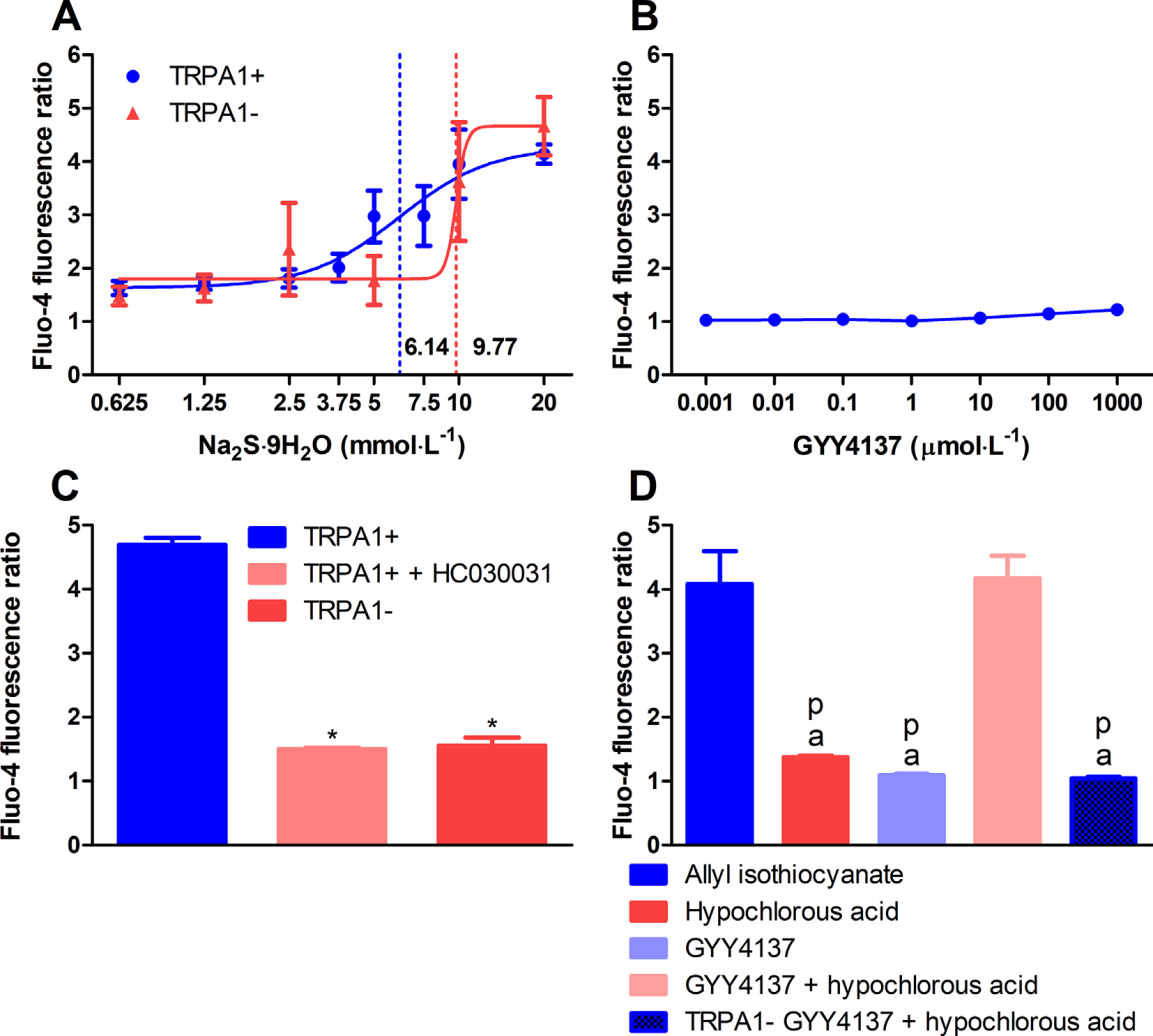
Polysulfide produced by hypochlorous acid out of sulfide released from acidified GYY4137 activates TRPA1 ion channels in CHO cells. **(A)** Sodium sulfide nonahydrate did not activate human TRPA1 ion channels expressed in CHO cells at physiological concentrations indicated by Fluo-4 AM fluorescence. EC_50_ values of TRPA1-expressing and non-expressing cells were 7.68 and 9.87 mmol L^−1^, respectively (*n* = 5–7, dotted lines show EC_50_ values). **(B)** GYY4137 did not elicit calcium influx in TRPA1-expressing CHO cells (*n* = 5). **(C)** Changes of [Ca^2+^]*_i_* of Fluo-4 AM-loaded CHO cells expressing human TRPA1 ion channels upon addition of sodium polysulfide (10 μmol L^−1^). Ca^2+^ concentration is characterized by increased fluorescence compared with dye-loaded unstimulated cells. Cell expressing human TRPA1 showed increased Ca^2+^ concentration in response to polysulfide. The reaction was prevented by treatment with HC-030031 (50 μmol L ^−1^) and lack of TRPA1 channels (*n* = 12, each data point represents 10^4^ cells. *p < 0.05vs. 10μmol L^−1^ of sodium polysulfide in TRPA1-expressing CHO cells). **(D)** Polysulfide produced by adding hypochlorous acid (616 μmol L^−1^) to acidified GYY4137 (7.95 mmol L^−1^ of GYY4137 plus equal volume of 1 mmol L^−1^ of hydrochloric acid) activates human TRPA1 ion channels expressed in CHO cells similarly to allyl isothiocyanate (100 μmol L ^−1^). Calcium influx was not detected in cells lacking the ion channel. Neither acidified GYY4137 nor hypochlorous acid alone induced calcium signals (*n* = 5–8, ^a^ *p* < 0.05 vs. 100 μmol L ^−1^ of allyl isothiocyanate, ^p^ *p* < 0.05 vs. acidified GYY4137 mixed with hypochlorous acid).

Sulfide was released from acidified solution of GYY4137. The GYY4137 solution released 328.1 ± 39.33 μmol L^−1^ of sulfide after 90-min incubation with citrate buffer (pH 3.0). Addition of 100 μmol L^−1^ of hypochlorous acid resulted in the formation of 77.15 ± 9.85 μmol L^−1^ of POLY (*n* = 18, data of six different experiments). Incubation of GYY4137 solution with equal volume of hydrochloric acid (1 mmol L^−1^) for 120 min resulted in the release of 147.3 ± 15.29 μmol L^−1^ of sulfide (*n* = 19, data of four different experiments). Treatment of CHO cells with sodium sulfide nonahydrate solution elicited calcium responses only at extremely supraphysiological concentrations. EC_50_ values in CHO cells expressing human TRPA1 ion channels and in ones lacking the channel were 6.14 and 9.77 mmol L^−1^, respectively (*n* = 5–7, data from four experiments; **Figure 2A**). No calcium influx was detected below 1 mmol L^−1^. Addition of GYY4137 to CHO cells with human TRPA1 up to 1 mmol L^−1^ did not induce any calcium signals (*n* = 5, data from four experiments; **Figure 2B**). POLY (10 μmol L^−1^) activated CHO cells expressing human TRPA1. TRPA1 activation by POLY was prevented by HC-030031 (50 μmol L^−1^) and was absent in cells not expressing the ion channel (*n* = 12, each data point represents ×10^4^ cells, **Figure 2C**). We investigated if POLY produced in GYY4137 solution acidified with hydrochloric acid (see above) by the addition of hypochlorous acid activates TRPA1 in CHO cells. Responses to TRPA1 agonist allyl isothiocyanate (100 μmol L^−1^) were used as positive control. Neither GYY4137 solution acidified with hydrochloric acid nor hypochlorous acid produced calcium influx compared with allyl isothiocyanate. On the other hand, the combination of acidified GYY4137 and hypochlorous acid induced a similar calcium response to that seen with allyl isothiocyanate. The calcium signal was absent if CHO cells not expressing TRPA1 ion channel were used (*n* = 5–8, **Figure 2D**).

### Sodium Polysulfide Stimulates Somatostatin Release From Nociceptor Nerve Endings in a TRPA1-Dependent Manner

**Figure 3:**
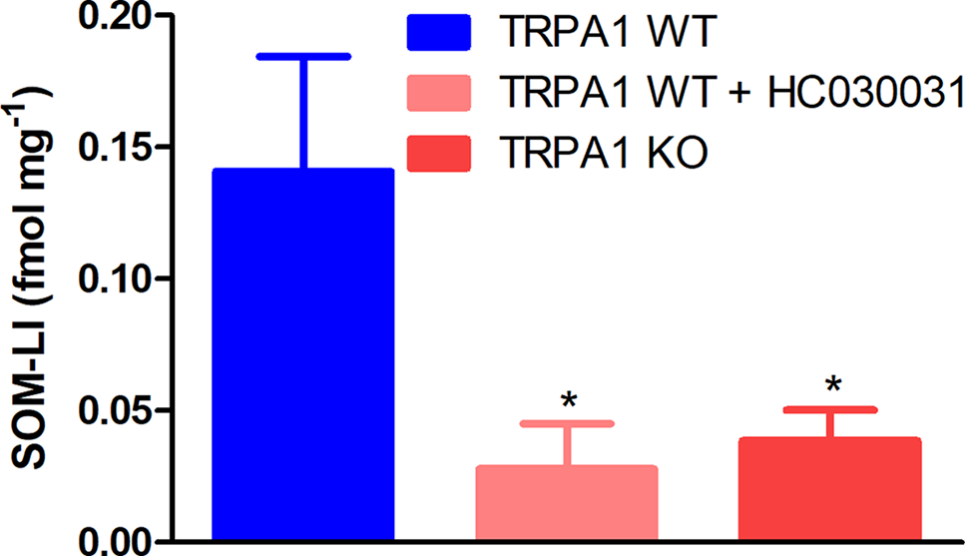
Sodium polysulfide (10 μmol L ^−1^) releases somatostatin from sensory nerve endings of isolated skin flaps of TRPA1 WT mice. Somatostatin release is inhibited by HC-030031 (50 μmol L ^−1^) and genetic lack of the ion channel (*n* = 12, *p < 0.05 vs. 10 μmol L ^−1^ of sodium polysulfide in TRPA1 WT samples).

Skin flaps of TRPA1 WT mice responded to stimulation with 10 μmol L^−1^ of POLY by releasing 0.1407 ± 0.044 fmol mg^−1^ of somatostatin-like immunoreactivity (SOM-LI). Treatment of the samples with 50 μmol L^−1^ of HC-030031 and genetic lack of TRPA1 ameliorated SOM liberation (0.1407 ± 0.044 vs. 0.0279 ± 0.017 and 0.0386 ± 0.012 fmol mg^−1^, *n* = 12, data from three separate experiments, **Figure 3**). Stimulation of skin samples with sodium sulfide nonahydrate up to 1 mmol L^−1^ did not provoke comparable SOM secretion (data are not shown).

### GYY4137 Aggravates Mechanical Hyperalgesia in Arthritic TRPA1 KO Animals and Ameliorates It in TRPA1 WT Animals

**Figure 4:**
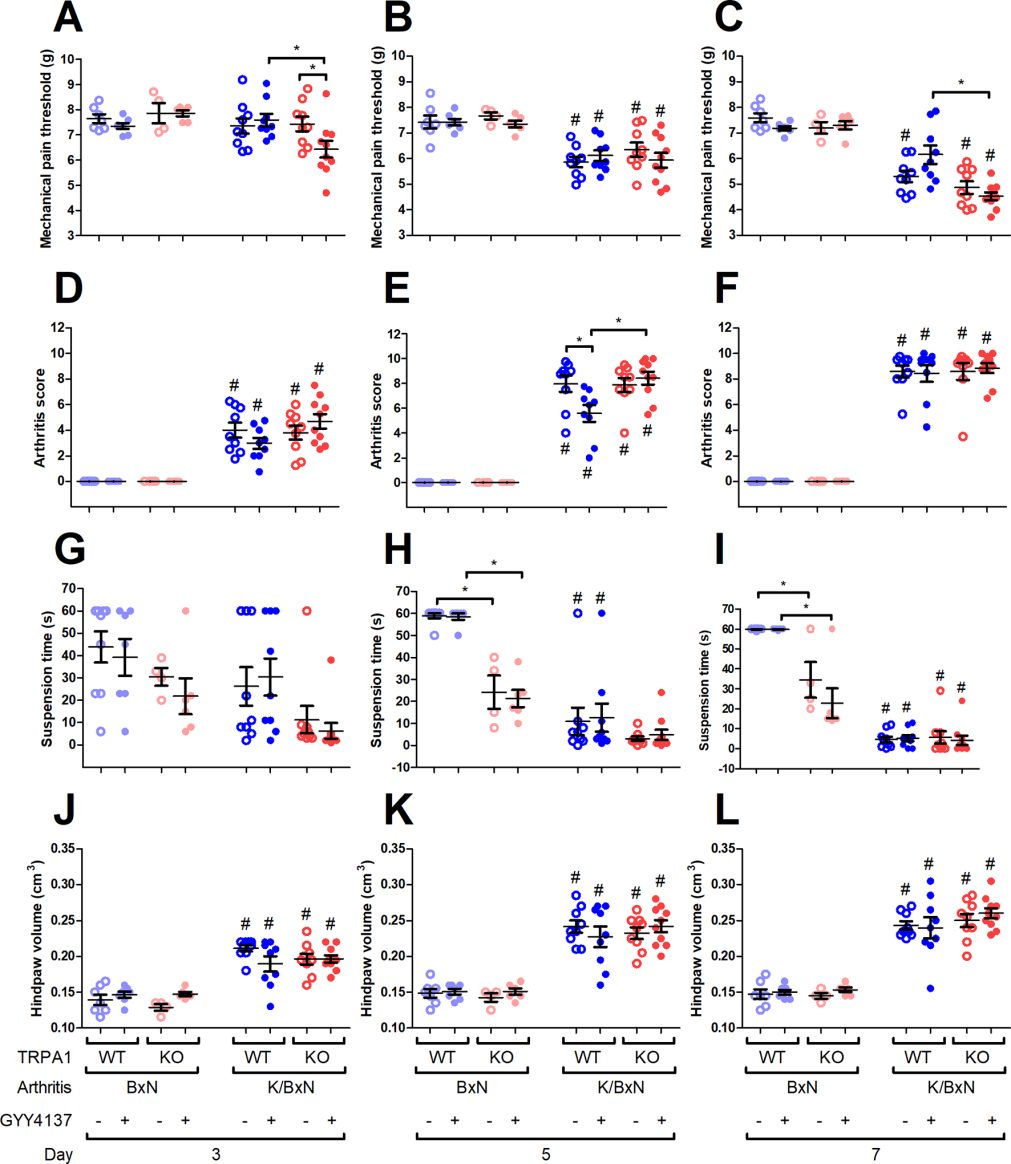
GYY4137 aggravates mechanical hyperalgesia of TRPA1 KO mice and ameliorates arthritis score of TRPA1 WT animals with K/BxN serum-transfer arthritis. Mechanical pain threshold of the hind paws (expressed in g) of GYY4137-treated (50 mg kg^−1^ day^−1^) and vehicle-treated TRPA1 WT and KO mice undergoing K/BxN arthritis on days **(A)** 3, **(B)** 5, and **(C)** 7. Semiquantitative clinical scores of GYY4137- and vehicle-treated TRPA1 WT and KO animals affected by serum-transfer arthritis on days **(D)** 3, **(E)** 5, and **(F)** 7. Time to failure of GYY4137- and vehicle-treated TRPA1 WT and KO mice having K/BxN arthritis in suspension test expressed in seconds on days **(G)** 3, **(H)** 5, and **(I)** 7. Hind paw volume detected by plethysmometry expressed in cm^3^ in GYY4137- and vehicle-treated TRPA1 WT and KO mice undergoing K/BxN arthritis on days **(J)** 3, **(K)** 5, and **(L)** 7. Whiskers show SEM; lines denote mean values, *n* = 9–10 arthritic mice per group and *n* = 6–9 non-arthritic mice per group. *p < 0.05 vs. indicated group. ^#^ *p* < 0.05 vs. BxN serum-treated animals.

K/BxN serum decreased the mechanical pain threshold in vehicle- or GYY4137-treated TRPA1 WT and KO mice (WT, BxN, vehicle treated, *n* = 7; WT, BxN, GYY4137 treated, *n* = 7; WT, K/BxN, vehicle treated, *n* = 9; WT, K/BxN, GYY4137 treated, *n* = 9; KO, BxN, vehicle treated, *n* = 9; KO, BxN, GYY4137 treated, *n* = 6; KO, K/BxN, vehicle treated, *n* = 9; WT, K/BxN, GYY4137 treated, *n* = 10) compared with the non-arthritogenic control mice (**Figures 4B, C**). On day 3, GYY4137 aggravated mechanical nociception in arthritic TRPA1 KO animals than did both vehicles of GYY4137 in TRPA1 KO animals and TRPA1 WT GYY4137-treated ones (6.43 ± 0.33 vs. 7.43 ± 0.29 g in TRPA1 KO vehicle treated and vs. 7.59 ± 0.25 g in TRPA1 WT GYY4137 treated; **Figure 4A**). On day 7, GYY4137 treatment ameliorated mechanical pain in TRPA1 WT animals compared with KO ones (6.15 ± 0.36 and 4.52 ± 0.15 g in GYY4137-treated TRPA1 WT and KO mice; **Figure 4C**). Neither GYY4137 nor genetic lack of TRPA1 altered mechanical pain threshold in animals injected with non-arthritogenic BxN serum (**Figures 4A–C**).

### GYY4137 Lowers Arthritis Score in TRPA1 WT Animals

Condition of the hind limbs was assessed based on a semiquantitative clinical scoring system (0–1.5, healthy; 1.5–2.5, minimal signs of disease; 2.5–4, mild inflammation; 4–7, moderate inflammation; 7–10, severe inflammation) described earlier by Jakus et al. (Jakus et al., 2010; Horváth et al., 2017). The condition of the hind limbs deteriorated in all K/BxN serum-treated mice (WT, BxN, vehicle treated, *n* = 7; WT, BxN, GYY4137 treated, *n* = 7; WT, K/BxN, vehicle treated, *n* = 9; WT, K/BxN, GYY4137 treated, *n* = 9; KO, BxN, vehicle treated, *n* = 9; KO, BxN, GYY4137 treated, *n* = 6; KO, K/BxN, vehicle treated, *n* = 9; WT, K/BxN, GYY4137 treated, *n* = 10; **Figures 4D–F**). The score of TRPA1 WT vehicle-treated mice undergoing arthritis was higher on day 5 than that of their GYY4137-treated counterparts (7.97 ± 0.66 vs. 5.58 ± 0.67; **Figure 4E**). The difference between GYY4137-treated TRPA1 WT and KO animals with serum-transfer arthritis stresses the beneficial effect of the drug in WT mice (5.58 ± 0.67 vs. 8.43 ± 0.51; **Figure 4E**). Neither GYY4137 nor TRPA1 KO genotype influenced arthritis score in mice injected with control BxN serum (**Figures 4D–F**).

### Genetic Lack of TRPA1 Impairs Hanging Performance in Mice Injected With BxN Serum Irrespective of GYY4137 Administration

The ability of arthritic mice to keep their suspended position was severely impaired in all groups regardless of genotype or treatment on day 7 and in TRPA1 WT animals on day 5 (WT, BxN, vehicle treated, *n* = 9; WT, BxN, GYY4137 treated, *n* = 7; WT, K/BxN, vehicle treated, *n* = 9; WT, K/BxN, GYY4137 treated, *n* = 9; KO, BxN, vehicle treated, *n* = 9; KO, BxN, GYY4137 treated, *n* = 6; KO, K/BxN, vehicle treated, *n* = 9; WT, K/BxN, GYY4137 treated, *n* = 10; **Figures 4H, I**). TRPA1 KO mice injected with inactive BxN serum exhibited statistically shorter suspension on days 5 and 7 when treated with vehicle than the respective TRPA1 WT groups (58.89 ± 1.11 vs. 20.3 ± 4.2 s and 58.57 ± 1.43 vs. 21.33 ± 3.93 s in non-arthritic TRPA1 WT vs. KO animals treated with vehicle on day 5; *p* < 0.001; 59.89 ± 0.11 vs. 25.74 ± 4.71 s and 59.86 ± 0.14 vs. 22.83 ± 7.45 s in BxN serum-injected TRPA1 WT vs. KO mice treated with vehicle on day 7; **Figures 4H, I**).

### GYY4137 Does Not Influence Body Weight and Hind Paw Volume Detected by Plethysmometry

Arthritis led to weight loss in all animal groups until day 7 (data are not shown). Neither differences of treatment nor those of genotype revealed any influence on the course of body weight changes. This was true as well in non-arthritic animals treated with BxN serum.

Swelling developed in the hind paws of all groups of arthritic mice (TRPA1 WT, BxN, vehicle treated, *n* = 7; TRPA1 WT, BxN, GYY4137 treated, *n* = 7; TRPA1 WT, K/BxN, vehicle treated, *n* = 9; TRPA1 WT, K/BxN, GYY4137 treated, *n* = 9; TRPA1 KO, BxN, vehicle treated, *n* = 9; TRPA1 KO, BxN, GYY4137 treated, *n* = 6; TRPA1 KO, K/BxN, vehicle treated, *n* = 9; TRPA1 WT, K/BxN, GYY4137 treated, *n* = 10; **Figures 4J–L**). Neither genetic constitution nor treatment with GYY4137 influenced paw swelling in K/BxN serum-injected arthritic and BxN-injected control mice (**Figures 4J–L**).

### Sst4 Receptors Do Not Mediate Protective Effects of GYY4137 in Serum-Transfer Arthritis

No differences in mechanical pain threshold, hanging performance, arthritis score, or body weight were found between GYY4137-treated sst4 WT and KO mice undergoing K/BxN arthritis. Similarly, no influence of the genetic lack of sst4 receptor was detected in arthritic mice receiving vehicle of GYY4137 (**Supplementary Figure 1**; WT, BxN, vehicle treated, *n* = 5; WT, BxN, GYY4137 treated, *n* = 5; WT, K/BxN, vehicle treated, *n* = 5; WT, K/BxN, GYY4137 treated, *n* = 6; KO, BxN, vehicle treated, *n* = 5; KO, BxN, GYY4137 treated, *n* = 5; KO, K/BxN, vehicle treated, *n* = 7; WT, K/BxN, GYY4137 treated, *n* = 9).

### GYY4137 Treatment Increases Neutrophil Granulocyte Accumulation and Plasma Extravasation in Arthritic TRPA1 KO Mice

**Figure 5:**
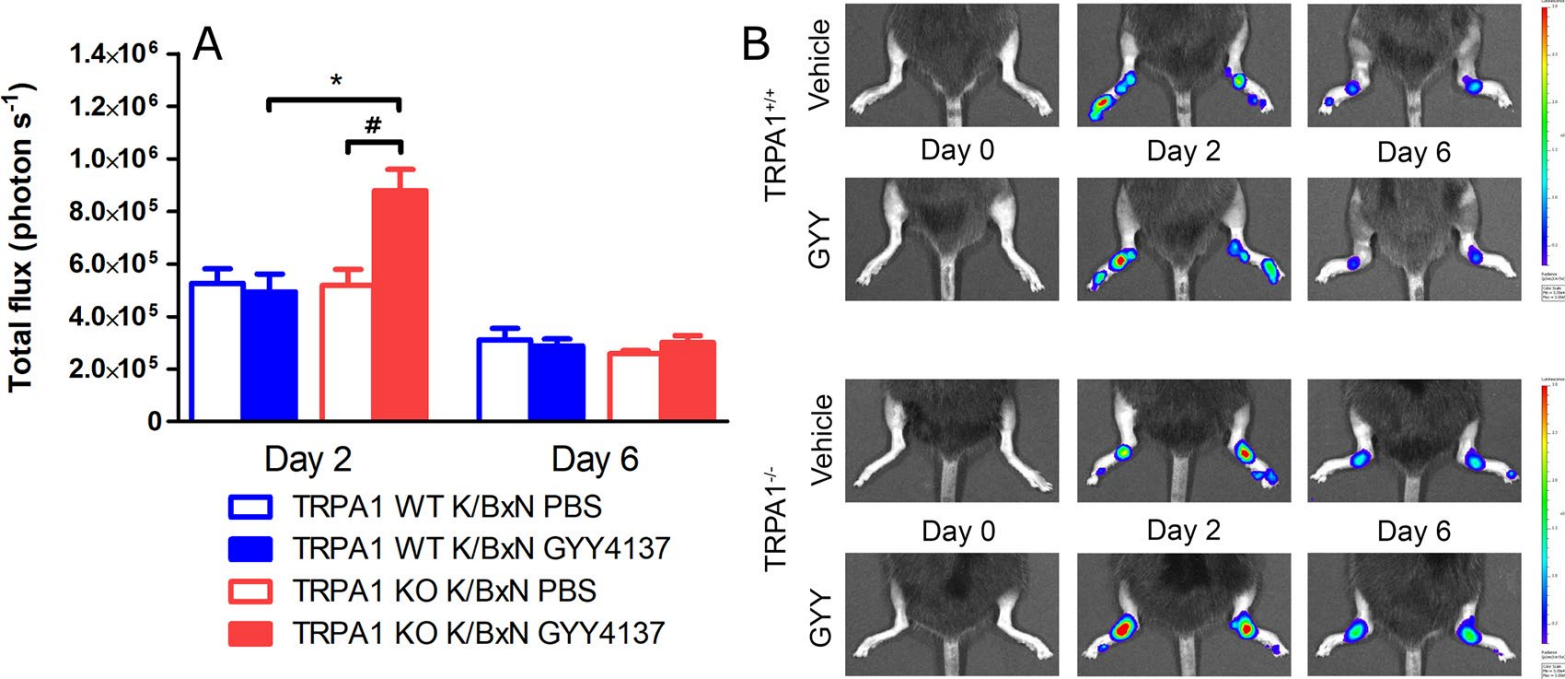
GYY4137 increases MPO activity in arthritic tibiotarsal joints of TRPA1 KO mice. **(A)** MPO activity in inflamed tibiotarsal joints of GYY4137-treated (50 mg kg^−1^day^−1^ i.p.) and vehicle-treated TRPA1 WT and KO animals undergoing K/BxN arthritis on days 2 and 6 shown as emitted photons s^−1^. **(B)** Representative bioluminescence images illustrating MPO activity characterized by luminol bioluminescence in tibiotarsal joints of GYY4137- and vehicle-treated TRPA1 WT and KO animals undergoing K/BxN arthritis. Data are shown as mean ± SEM of *n* = 9–10 mice per group. *p < 0.05 vs. GYY4137-treated TRPA1 WT mice. ^#^ *p* < 0.05 vs. TRPA1 KO animals treated with vehicle of GYY4137.

**Figure 6:**
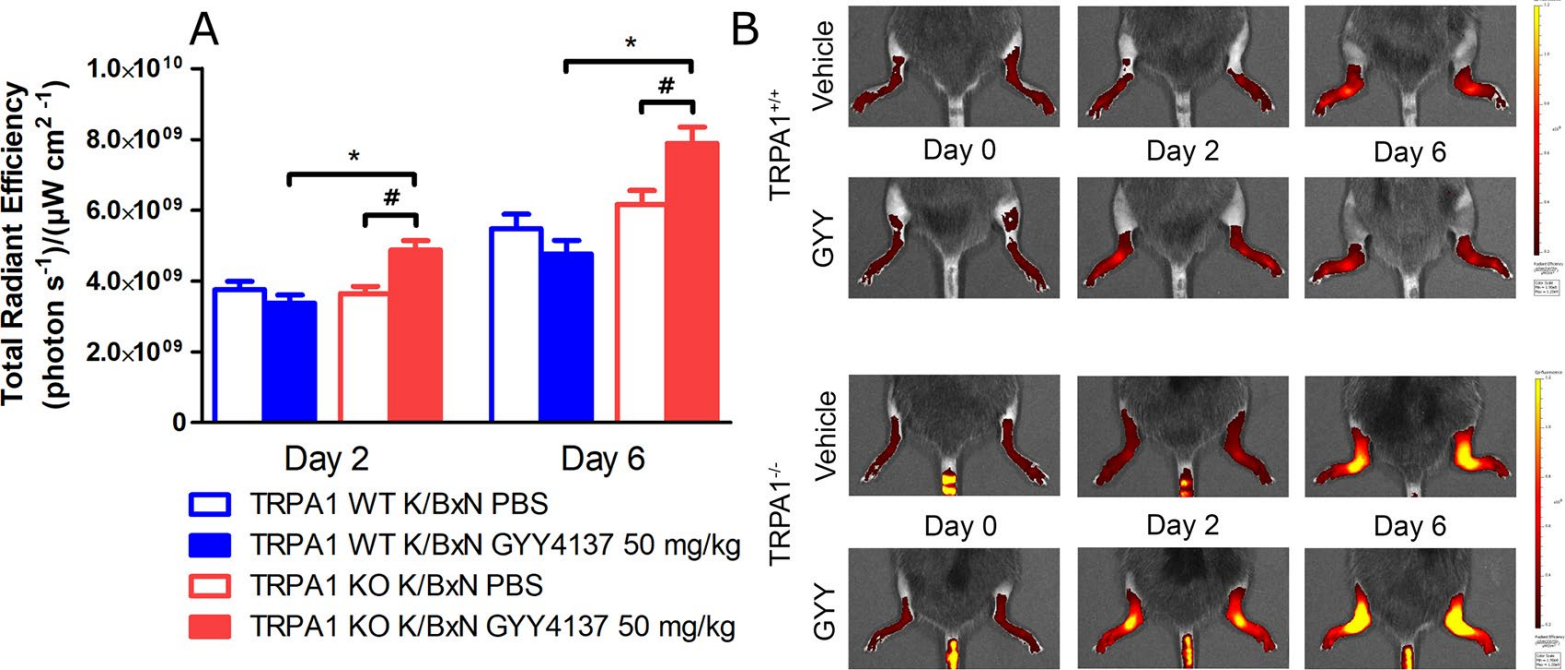
GYY4137 exacerbates plasma extravasation in arthritic tibiotarsal joints of TRPA1 KO mice. **(A)** Edema formation characterized by extravasation of micellar fluorescent IR-676 dye in tibiotarsal joints of GYY4137-treated (50 mg kg^−1^day^−1^i.p.) and vehicle-treated TRPA1 WT and KO mice undergoing K/BxN arthritis on days 2 and 6 expressed as (photons s^−1^)(μW cm^−2^)^−1^. **(B)** Representative fluorescence images showing plasma protein extravasation characterized by extravasation of micellar IR-676 dye in tibiotarsal joints of GYY4137- and vehicle-treated TRPA1 WT and KO mice undergoing K/BxN arthritis. Data are shown as mean ± SEM of *n* = 9–10 mice per group. *p < 0.05 vs. GYY4137-treated TRPA1 WT mice. ^#^ *p* < 0.05 vs. TRPA1 KO animals treated with vehicle of GYY4137.

Neutrophil cell accumulation characterized by luminol bioluminescence indicating MPO activity was measured before serum transfer and then 2 and 6 days later. Daily treatment with GYY4137 elevated bioluminescence at day 2 in TRPA1 KO animals compared with TRPA1 WT GYY4137-treated mice and with TRPA1 KO vehicle-treated ones as well (*n* = 9–10 mice; 8.79 10^5^ ± 8.03 10^4^ vs. 4.94 10^5^ ± 6.74 10^4^ and 5.19 10^5^ ± 6.04 10^4^ photons s^−1^; **Figures 5A, B**). These data suggest that H_2_S aggravates accumulation of neutrophil granulocytes in TRPA1 KO animals at day 2—when the involvement of these cells is maximal in the arthritic reaction—but does not influence cellular inflammation in WT mice. At day 6, there is no more participation of neutrophil cells in the foreground of serum-transfer arthritis. Plasma extravasation detected as IR-676 fluorescence in tissues was aggravated by GYY4137 injections in TRPA1 KO animals in relation to TRPA1 WT GYY4137-treated mice and to TRPA1 KO vehicle-treated animals at both 2 days (*n* = 9–10 mice; 4.87 10^9^ ± 2.73 10^8^ vs. 3.37 10^9^ ± 2.3 10^8^ and 3.65 10^9^ ± 2 10^8^ (photons s^−1^)(ခμW cm^−2^)^−1^; **Figures 6A, B**) and 6 days (*p* < 0.001 and *p* < 0.01; 7.89 10^9^ ± 4.58 10^8^ vs. 4.76 10^9^ ± 3.89 10^8^ and 6.16 10^9^ ± 3.91 10^8^ (photons s^−1^)(ခμW cm^−2^)^−1^; **Figures 6A, B**). According to our findings, the rate of ongoing plasma extravasation—detected by fluorescence imaging—was elevated by H_2_S in TRPA1 KO animals at both days 2 and 6 but was left unaffected in WT ones.

### Genetic Lack of sst4 Somatostatin Receptor Does Not Influence Myeloperoxidase Enzyme Activity and Plasma Extravasation in Arthritic Mice

No difference in MPO activity detected by luminescence imaging was recorded between arthritic sst4 receptor WT and KO GYY4137-treated animals. No participation of the receptor showed up when comparing sst4 WT and KO mice receiving vehicle of GYY4137. Similar data were generated when detecting plasma extravasation by fluorescence imaging (**Supplementary Figure 2**; WT, BxN, vehicle treated, *n* = 5; WT, BxN, GYY4137 treated, *n* = 5; WT, K/BxN, vehicle treated, *n* = 5; WT, K/BxN, GYY4137 treated, *n* = 6; KO, BxN, vehicle treated, *n* = 5; KO, BxN, GYY4137 treated, *n* = 5; KO, K/BxN, vehicle treated, *n* = 7; WT, K/BxN, GYY4137 treated, *n* = 9).

### GYY4137 Elevates MIP-2 Level in Inflamed Hind Paws of TRPA1 KO Mice

**Figure 7:**
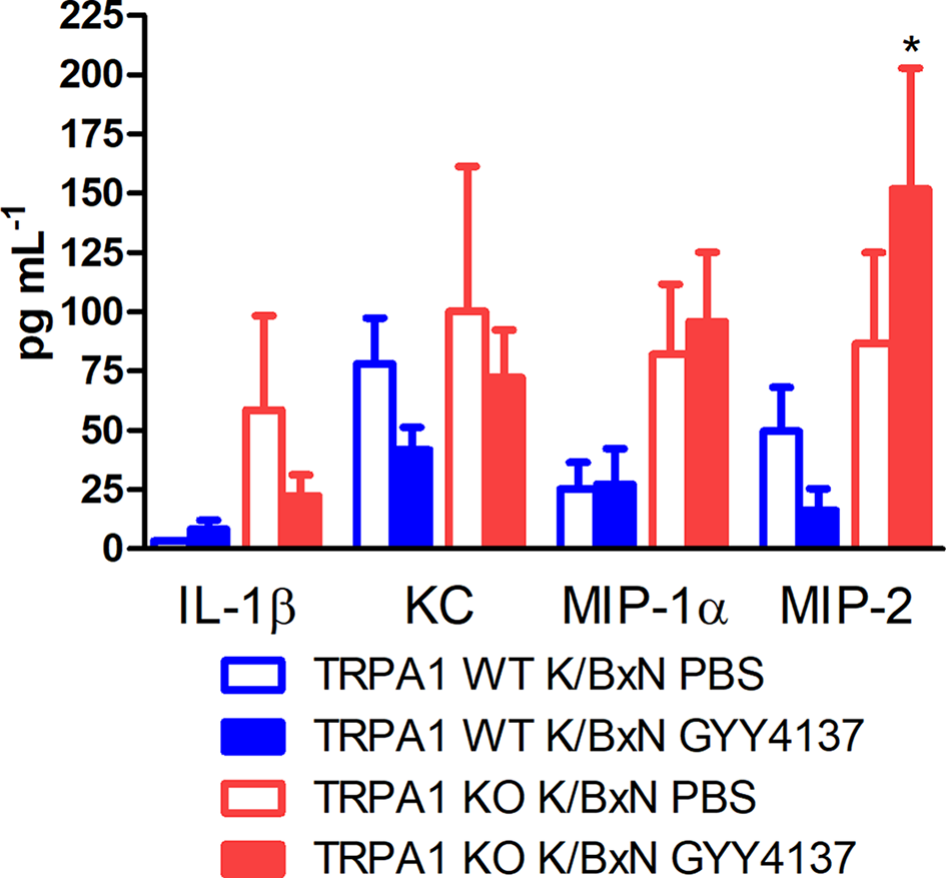
GYY4137 elevates MIP-2 concentration in inflamed paws of TRPA1 KO mice. Concentrations of IL-1β, KC, MIP-1α and MIP-2 in lavage fluid of the subcutaneous tissue of the inflamed hind paws of GYY4137-treated (50 mg kg^−1^ day ^−1^ i.p.) and vehicle-treated TRPA1 WT and KO mice undergoing K/BxN arthritis measured at day 3. Data are mean ± SEM of *n* = 6–8 mice per group, *p < 0.05 vs. GYY4137-treated TRPA1 WT animals.

Serum-transfer arthritis did not affect concentrations of IL-1β, KC and MIP-1α in the subcutaneous flushing fluid of the hind paws (*n* = 12–16). In case of MIP-2, larger concentrations were measured in TRPA1 KO GYY4137-treated animals compared with WT GYY4137-treated ones—despite remarkably large standard deviation of data (16.31 ± 9.14 vs. 151.8 ± 50.93 pg mL^−1^ in TRPA1 WT and KO GYY4137-treated mice; **Figure 7**).

### GYY4137 Ameliorates Histological Cartilage Destruction in TRPA1 Wild-Type Animals

**Figure 8:**
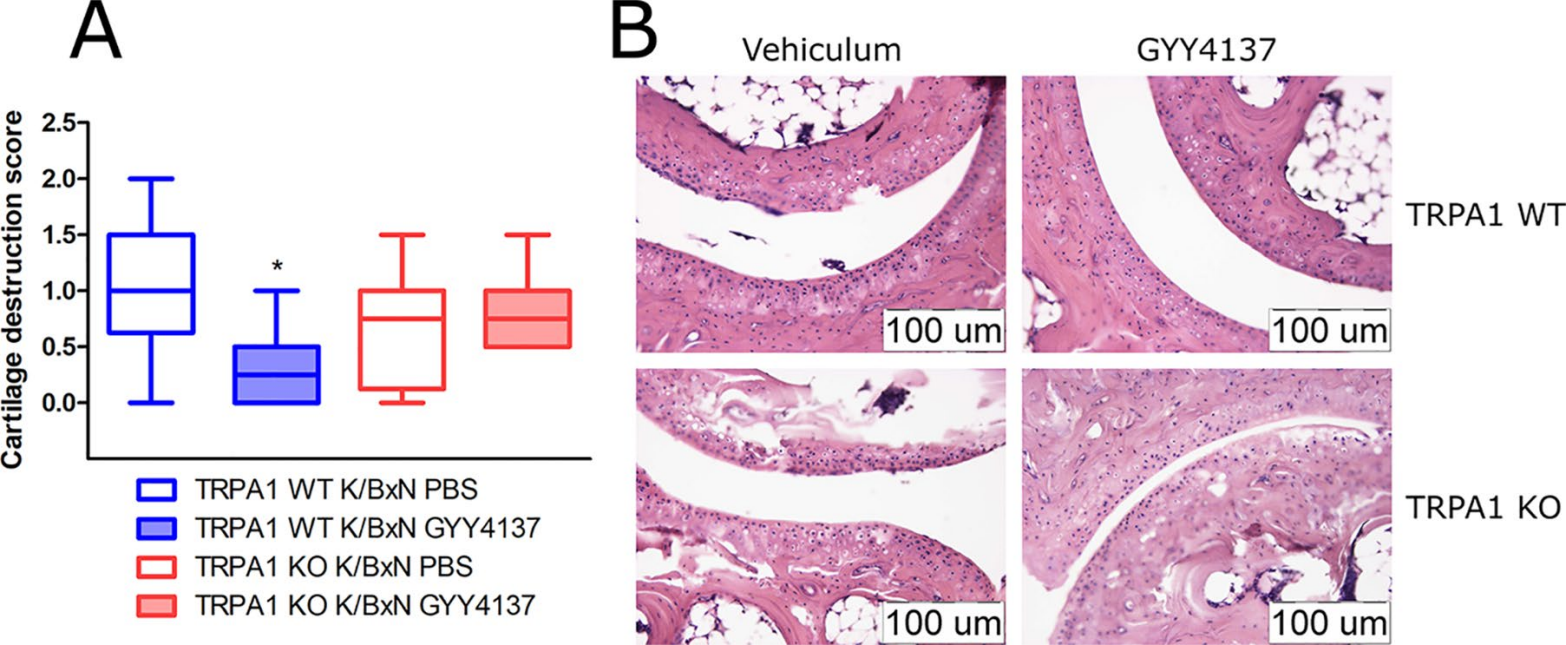
GYY4137 decreases cartilage destruction in tibiotarsal joints of arthritic TRPA1 WT mice. **(A)** Cartilage destruction score of tibiotarsal joints of GYY4137-treated (50 mg kg^−1^day^−1^ i.p.) and vehicle-treated TRPA1 WT and KO mice undergoing K/BxN arthritis. Samples were taken on day 7. Boxes range between the 25th and 75th percentiles, horizontal lines denote medians, and whiskers extend from minimal to maximal values. *n* = 8, *p < 0.05 vs. TRPA1 WT animals treated with vehicle of GYY4137. Kruskal–Wallis test followed by Dunn’s test. **(B)** Representative histological images illustrating damage of cartilage in hematoxylin and eosin-stained slides of vehicle- or GYY4137-treated TRPA1 WT and KO animals injected with K/BxN serum. Images were taken at a 20× magnification.

Cartilage destruction score was lowered by GYY4137 (50 mg kg^−1^ day^−1^ i.p.) in TRPA1 WT arthritic mice compared with vehicle-treated ones (1.063 ± 0.22 vs. 0.3125 ± 0.13, *n* = 8, **Figures 8A, B**). No difference of cartilage damage was noted in TRPA1 KO animals. Other histological parameters (mononuclear cell infiltration, synovial hyperplasia, fibroblast accumulation and collagen deposition, and the composite score) did not change in vehicle- or GYY4137-treated or animals of any genotype. Sst4 receptor WT animals treated with GYY4137 exhibited elevated fibroblast cell count and collagen deposition in comparison to vehicle-treated ones (**Supplementary Figure 3**).

## Discussion

The main novel finding of this study is that the presence or absence of functional TRPA1 channels determines pronociceptive, pro-inflammatory, or, conversely, analgesic and anti-inflammatory effects of sulfide. Mice undergoing K/BxN serum-transfer arthritis and lacking functional TRPA1 receptors showed more severe mechanical hyperalgesia, more expressed plasma extravasation, and MPO activity in the inflamed limb, as well as increased MIP-2 concentration in the affected paws when administered GYY4137. Alternatively, the slow-releasing sulfide donor ameliorated hyperalgesia, arthritis score, and histological cartilage destruction in TRPA1 WT animals. Another interesting point is that the protective effect was not mediated directly by sulfide itself but by polysulfides being formed out of it.

The protective role of TRPA1-mediated GYY4137 effect is pronounced by DPA measurement. WT animals showed elevated mechanical pain threshold, but we detected aggravated reaction in KO mice. K/BxN serum proved to be arthritogenic in all examined genotypes. GYY4137 caused significant reduction of the arthritis on day 5 in TRPA1 WTs. Interestingly, in our study, TRPA1 KO animals injected with non-arthritogenic BxN serum exhibited shorter time to failure than did their WT counterparts when suspended upside-down irrespective of GYY4137 or vehicle administration. Muscle cramps of athletes upon isometric strain might resemble a similar situation. TRPA1 and transient receptor potential vanilloid 1 (TRPV1) agonists prevent such cramps, supporting our findings (Craighead et al., 2017). Lee and colleagues reported on deteriorated motor performance of TRPA1 KO mice (Lee et al., 2017).

In the hands of other authors, GYY4137 had an anti-inflammatory action in CFA-induced murine arthritis. GYY4137 inhibited mediator release from activated human synoviocytes and articular chondrocytes as well (Fox et al., 2012; Li et al., 2013). These previous data are in concordance with our present results: TRPA1 WT mice showed milder mechanical hyperalgesia, arthritis score, and cartilage damage in response to GYY4137.

Surprisingly, GYY4137 did not affect paw swelling measured by plethysmometry but increased plasma extravasation detected by fluorescence imaging of IR-676 in TRPA1 KO animals. Plethysmometry was executed on days 3, 5, and 7; fluorescence imaging was performed on days 2 and 6. The two methods detect different parameters. Plethysmometry provides information about the cumulative swelling—which means the equilibrium of edema formation and resolution—since the initiation of inflammation. Fluorescence imaging characterizes the actual rate or velocity of plasma extravasation since the administration of the fluorescent dye (20 min). The other seemingly conflicting data are the elevated MPO activity and MIP-2 content of inflamed paws despite no change in histological mononuclear cell accumulation score. MPO activity and cytokine measurements were performed on days 2 and 3, while histological evaluation took place at a different stage of the inflammatory process on day 7. At this time, rather, mononuclear cells dominate, and neutrophil accumulation together with MPO activity ceases.

It might seem also contradictory how the activation of TRPA1 channels and an increase of [Ca^2+^]*_i_* in nociceptors could lead to an opposite effect than nociception and inflammation. One proposed mechanism is the release of inhibitory neuropeptides (e.g., somatostatin). SOM release might be due to opening of TRPA1 or TRPV1 channels (Pethő et al., 2017; Pozsgai et al., 2017). Elevated SOM plasma concentration in response to painful disease was detected in rodents and patients (Helyes et al., 2004; Antal et al., 2008; Suto et al., 2010). SOM possesses systemic antinociceptive and anti-inflammatory properties mediated by sst_4_ receptors (Helyes et al., 2004; Pintér et al., 2006; Schuelert et al., 2015; Pozsgai et al., 2017). Sst4 receptors expressed in sensory neurons, lymphocytes, and vascular endothelial cells might contribute to the protective effect (Pintér et al., 2006). However, in the present model of arthritis, genetic lack of sst4 receptors did not diminish protective effect of GYY4137. It has to be noted that protective effect of GYY4137 on mechanical pain threshold was also not detected in sst4 WT mice. This might be explained by minor genetic differences between TRPA1 and sst4 genetically modified mouse strains together with compensatory changes of gene expression in genetically modified animals. Macrophages play a decisive role in the mediation of serum-transfer arthritis (Christensen et al., 2016). Somatostatin sst2 receptors dominate in human monocytes and macrophages (Dalm et al., 2003). Sst2 receptor agonist octreotide inhibits release of inflammatory cytokines (TNF-α, IL-6, and IL-15) from synoviocytes of RA patients, offering a plausible mechanism for the anti-inflammatory effect of GYY4137 (Casnici et al., 2018). Besides, sensory neuron-derived opioid peptides might contribute to antinociceptive action, too (Pethő et al., 2017).

The protective effect of sulfide in the K/BxN serum-transfer arthritis model might be mediated by non-neuronal TRPA1 expressed on immune and inflammatory cells. CD4+ T cells have a major role in the development of serum-transfer arthritis, as mutation of the T-cell receptor and formation of autoreactive T cells are key features of the model. Depletion of either neutrophil granulocytes or macrophages is protective against serum-transfer arthritis (Christensen et al., 2016). TRPA1 expression in CD4+ T cells was confirmed on the level of mRNA and by immunohistochemistry. TRPA1 channels of T lymphocytes were found to be functional by electrophysiological methods. Genetic abrogation of TRPA1 exacerbated a murine model of autoimmune colitis. TRPA1 was found to inhibit Th1 immune response (Bertin et al., 2017). TRPA1 was detected in murine CD4+ T cells in a model of psoriasis by immunohistochemistry (Kemény et al., 2018). Activation of the ion channel mediates a protective effect in psoriasiform dermatitis. Increased expression of TRPA1 was reported in peripheral blood leukocytes of rheumatoid arthritis patients. Antagonism of TRPA1 aggravated inflammation and MIP-2 secretion from macrophages. TRPA1 agonist allyl isothiocyanate was protective against atherosclerosis and ameliorated MIP-2 release (Zhao et al., 2016). All the above findings indicate that activation of TRPA1 by sulfide on T lymphocytes, neutrophil granulocytes, or macrophages might have contributed to the anti-inflammatory effect revealed by the present study.

Several possible mechanisms could be supposed in the background of TRPA1-independent pro-inflammatory effect of GYY4137. Sulfide donor NaHS produced non-canonical p38/Akt and CREB activation in human peripheral blood monocytes (Sulen et al., 2016). Various inflammatory animal models were reported to be augmented by sulfide or alleviated by disabled synthesis of the gasotransmitter. The signaling pathways involved include PI3K/Akt/Sp1, COX2, and NF-κB (Ang et al., 2011; Badiei et al., 2016; Gaddam et al., 2016; George et al., 2016; Liu et al., 2016; Ahmad et al., 2017; Liu et al., 2017). Our results indicate that TRPA1-mediated protective effects of H_2_S “overwrite” inflammatory and pro-algesic effects mediated via other pathways. Detrimental effects only manifest in the absence of TRPA1.

According to our data, concentration of the chemoattractant MIP-2 (CXCL2) was elevated by GYY4137 in inflamed hind paws of arthritic TRPA1 KO animals on day 3. This finding is in alignment with increased MPO activity—denoting neutrophil granulocyte accumulation—in tibiotarsal joints of TRPA1 KO mice detected on day 2. MIP-2 is a chemoattractant for neutrophil cells and is released by monocytes, macrophages, epithelial cells, and hepatocytes (Qin et al., 2017). In the K/BxN serum-transfer arthritis model, activated neutrophils might express CXCL2 themselves. Recruitment of neutrophil granulocytes depends on the expression of CXCR2, the receptor of MIP-2. Depletion of macrophages—an important source of MIP-2—is protective against serum-transfer arthritis (Christensen et al., 2016). Activation of TRPA1 is involved in MIP-2 secretion, too. TRPA1 KO mice showed lowered concentration of the chemoattractant in a subcutaneous air pouch model (Moilanen et al., 2015).

Our data point towards GYY4137 being an “inflammation-selective” polysulfide donor. As it was described earlier and confirmed by our data, sulfide release from GYY4137 increases dramatically at pH 3.0 (Li et al., 2008). Acidic pH was found in RA synovial fluid (Martínez et al., 2006). Addition of hypochlorous acid to the liberated sulfide rapidly yields POLY (Nagy and Winterbourn, 2010). We postulate that excess sulfide released in acidic inflamed tissues is scavenged by hypochlorite from neutrophil granulocytes. Lack of hypochlorite was suspected to increase MIP-2 production of neutrophil cells (Tateno et al., 2013). Elevated MIP-2 concentrations were measured in subcutaneous flushing fluid of arthritic TRPA1 KO mice by the authors. On the other hand, POLY resulting from the reaction of sulfide and hypochlorite persulfidates TRPA1 ion channels of nociceptor nerve endings and might trigger protective effects. Production of hypochlorite by neutrophils is MPO dependent (Albrett et al., 2018). Elevated MPO activity in GYY4137-treated TRPA1 KO animals suggests larger hypochlorite and POLY formation. A larger amount of POLY could mediate more expressed TRPA1-independent inflammatory changes.

Inconsistent data on the effect of sulfide in inflammation and nociception have been published since the advent of sulfide research a decade ago. According to our present study, protective actions of the gasotransmitter are associated with TRPA1 receptor activation, while detrimental ones are mediated by other mechanisms in murine arthritis. In the present study, slow-releasing sulfide donor GYY4137 is an instrument to study the role of TRPA1 ion channel in the effects of sulfide in murine K/BxN serum-transfer arthritis model. Our approach provides hints on the importance of TRPA1 channels for the development and application of sulfide therapeutics depending on whether amelioration of inflammation or cytotoxic effect is desired.

## Data Availability

All datasets generated and analyzed for this study are included in the manuscript and/or the **Supplementary Files**.

## Ethics Statement

This study was carried out in accordance with the recommendations of 1998/XXVIII Act of the Hungarian Parliament on Animal Protection and Consideration Decree of Scientific Procedures of Animal Experiments (243/1998) and the European Communities Council Directive of 2010/63/EU, complied with the recommendations of the International Association for the Study of Pain (IASP). The protocol was approved by the Ethics Committee on Animal Research of University of Pécs and Semmelweis University according to the Ethical Codex of Animal Experiments (licence No.: BA 02/2000–2/2012 and PE/EA/3895-6/2016).

## Author Contributions

ÁH, GP, ZH, and EP designed the study. IB, ÁH, and GP conducted experiments. IB, BN, and ZS performed fluorescent Ca^2+^ imaging experiments on CHO cells. ÁH and IB performed IVIS measurements. CS synthesized GYY4137. KB optimized experiments on SOM release from murine skin. ÉB and AP performed histology and evaluation of slides. ÁK performed cytokine measurements. AM provided BxN and K/BxN sera and corresponding know-how. ZH, AM, GP, and EP drafted the manuscript. All authors have read and approved the final manuscript.

## Funding

This project was supported by the János Bolyai Research Scholarship (GP) and by János Szentágothai Fellowship A2-SZJÖ-TOK-13-0149 (EP) of the Hungarian Academy of Sciences. This work was funded by grants GINOP-2.3.2.-15-2016-00048 STAY ALIVE and EFOP-3.6.2-16-2017-00006 LIVE LONGER from the European Regional Development Fund. The study was supported by the ÚNKP-18-4 New National Excellence Program of the Ministry of Human Capacities. BN was supported by Richter Gedeon Talentum Alapítvány.

## Conflict of Interest Statement

The authors declare that the research was conducted in the absence of any commercial or financial relationships that could be construed as a potential conflict of interest.
