# Supplementary material for: TRPA1 Ion Channel Determines Beneficial and Detrimental Effects of GYY4137 in Murine Serum-Transfer Arthritis
Source: Front Pharmacol. 2019 Sep 4;10:964. doi: 10.3389/fphar.2019.00964 (PMC6737045; doi:10.3389/fphar.2019.00964)
Supplement: Supplementary file 1 [file DataSheet_1.pdf]

Supplementary material

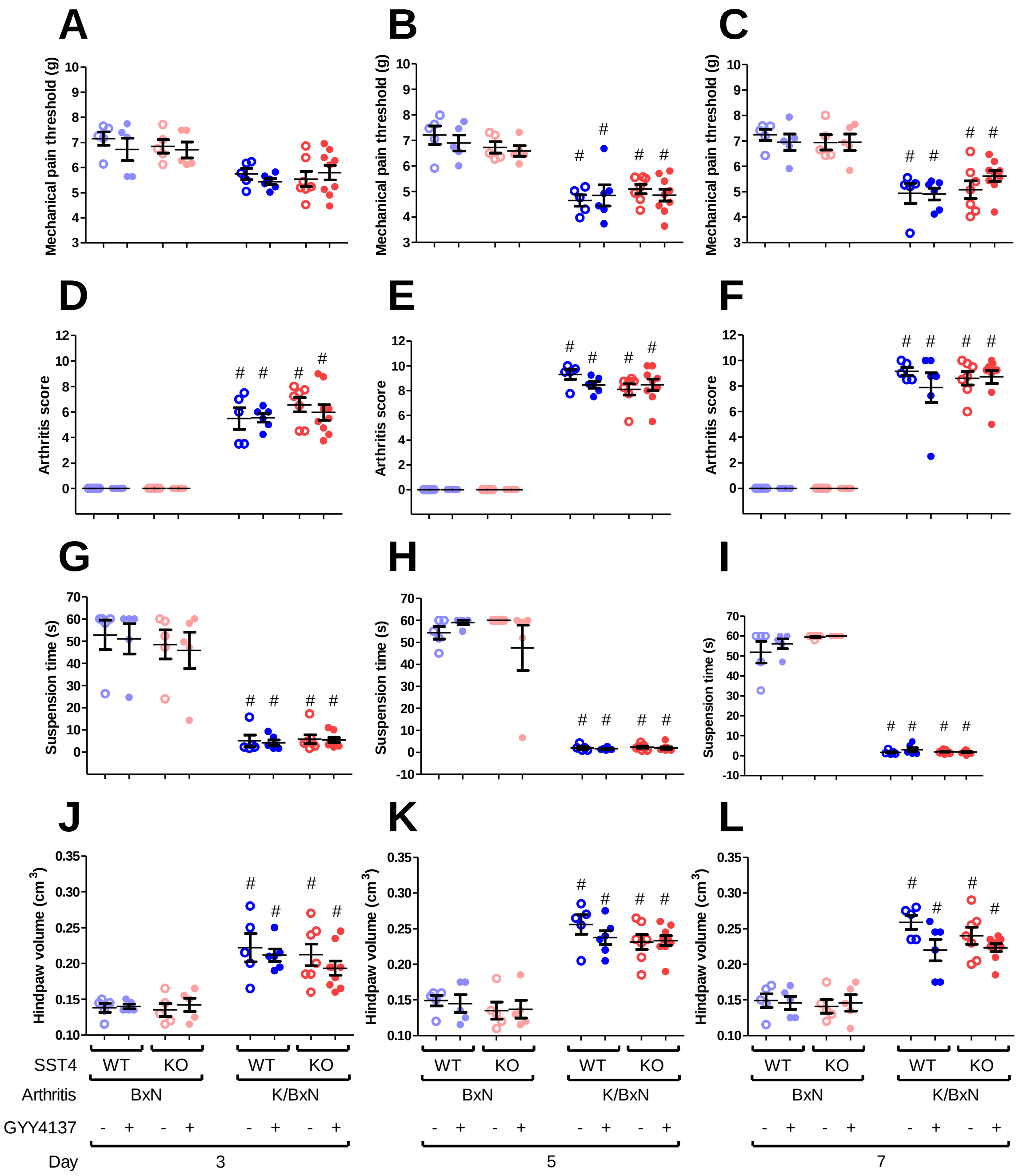

Supplementary figure 1. GYY4137 treatment had no influence on serum-transfer arthritis in sst4 WT and KO mice. Mechanical pain threshold of the hind paws (expressed in g) of GYY4137- (50 mg/kg/day) and vehicle-treated sst4 WT and KO mice undergoing K/BxN arthritis on days (A) 3, (B) 5 and (C) 7. Semiquantitative clinical scores of GYY4137- and vehicle-treated sst4 WT and KO animals affected by serum-transfer arthritis on days (D) 3, (E) 5 and (F) 7. Time to failure of GYY4137- and vehicle-treated

sst4 WT and KO mice having K/BxN arthritis in suspension test expressed in seconds on days (G) 3, (H) 5 and (I) 7. Hind paw volume detected by plethysmometry expressed in cm^3 in GYY4137- and vehicle-treated sst4 WT and KO mice undergoing K/BxN arthritis on days (J) 3, (K) 5 and (L) 7. Whiskers show SEM, lines denote mean values, n = 5-9 arthritic mice/group and n = 5 non-arthritic mice/group. # p < 0.05 vs. BxN serum-treated animals. One-way ANOVA followed by Tukey's multiple comparison test.

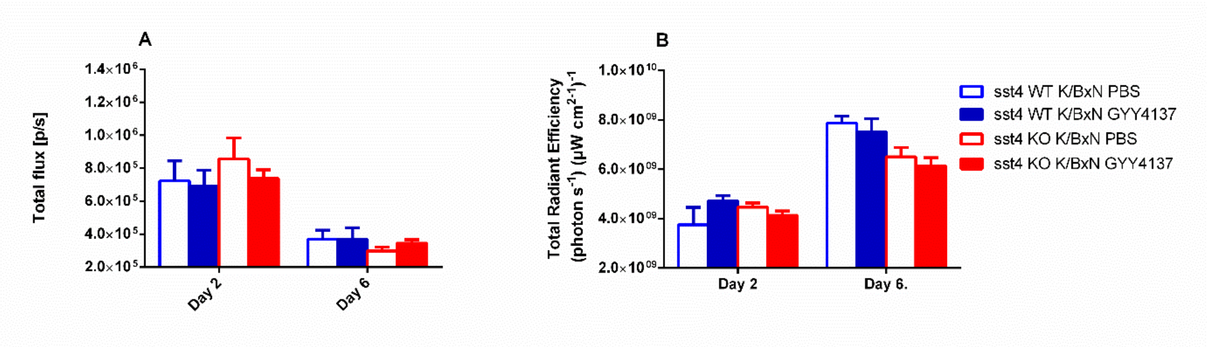

Supplementary figure 2. No changes were observed in MPO activity and plasma extravasation in arthritic tibiotarsal joints of sst4 WT and KO animals in response to GYY4137 or vehicle treatment. MPO activity in inflamed tibiotarsal joints of GYY4137- (50 mg/kg/day i.p.) and vehicle-treated sst4 WT and KO animals undergoing K/BxN arthritis on days 2 and 6 shown as emitted photons s^{-1}. (A). Edema formation characterized by extravasation of micellar fluorescent IR-676 dye in tibiotarsal joints of GYY4137- (50 mg/kg/day i.p.) and vehicle-treated sst4 WT and KO mice (B). Data are shown as mean ± SEM of n=5-9 mice/group. One-way ANOVA followed by Tukey's multiple comparison test.

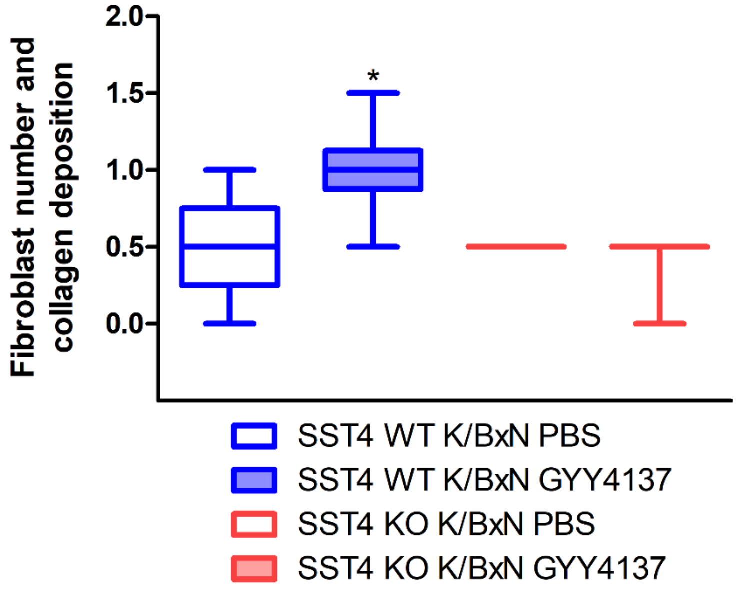

Supplementary figure 3. GYY4137 increased fibroblast cell count and collagen deposition score in sst4 WT arthritic mice. Samples were taken on day 7. Boxes range between percentiles 25 and 75, horizontal lines denote medians and whiskers extend from minimal to maximal values. n =5-9. Kruskal-Wallis test followed by Dunn’s test.
